# Polyphenols as Modulators of Aquaporin Family in Health and Disease

**DOI:** 10.1155/2015/196914

**Published:** 2015-08-04

**Authors:** Diana Fiorentini, Laura Zambonin, Francesco Vieceli Dalla Sega, Silvana Hrelia

**Affiliations:** ^1^Department of Pharmacy and Biotechnology, Alma Mater Studiorum, University of Bologna, Via Irnerio 48, 40126 Bologna, Italy; ^2^Department for Life Quality Studies, Alma Mater Studiorum, University of Bologna, Corso Augusto 237, 47921 Rimini, Italy

## Abstract

Polyphenols are bioactive molecules widely distributed in fruits, vegetables, cereals, and beverages. Polyphenols in food sources are extensively studied for their role in the maintenance of human health and in the protection against development of chronic/degenerative diseases. Polyphenols act mainly as antioxidant molecules, protecting cell constituents against oxidative damage. The enormous number of polyphenolic compounds leads to huge different mechanisms of action not fully understood. Recently, some evidence is emerging about the role of polyphenols, such as curcumin, pinocembrin, resveratrol, and quercetin, in modulating the activity of some aquaporin (AQP) isoforms. AQPs are integral, small hydrophobic water channel proteins, extensively expressed in many organs and tissues, whose major function is to facilitate the transport of water or glycerol over cell plasma membranes. Here we summarize AQP physiological functions and report emerging evidence on the implication of these proteins in a number of pathophysiological processes. In particular, this review offers an overview about the role of AQPs in brain, eye, skin diseases, and metabolic syndrome, focusing on the ability of polyphenols to modulate AQP expression. This original analysis can contribute to elucidating some peculiar effects exerted by polyphenols and can lead to the development of an innovative potential preventive/therapeutic strategy.

## 1. Introduction

Aquaporins (AQPs) are integral, transmembrane, small hydrophobic water channel proteins. To date, the family consists of 13 members, highly conserved across the plant and animal kingdoms. The major AQP function is to facilitate the transport of water over cell plasma membranes; some members of AQP family are also able to transport other small molecules, such as glycerol, urea, CO_2_, ammonia, and nitric oxide (for a review see [1]). AQPs play a prominent role in regulating physiological functions of many organs and tissues, and their functions have mainly been studied in the brain, kidney, glands, and skeletal muscle. Emerging evidence suggests that expression of these proteins is altered in mammary tumors and in cancer cell lines and has been implicated in a number of pathophysiological processes. This review contextualizes the importance of AQPs in brain, eye, skin diseases, and metabolic syndrome, focusing the analysis on AQP modulation by polyphenols, bioactive molecules widely present in food sources and extensively studied for their role in the prevention of degenerative diseases and in the maintenance of human health.

Each polyphenolic compound, or class of polyphenols, will be introduced and extensively described in the relevant section.

## 2. AQP Structure and Substrates

The first characterization of AQP, now named as AQP1, was performed in 1992 by Preston et al., who functionally described the protein from human red blood cells [2]. He demonstrated that the presence of a transmembrane channel protein could explain the high membrane water permeability of erythrocytes and other cells, which could not be justified only by simple passive diffusion of water molecules across the lipid bilayers [3]. Currently, it is known that AQP expression on cell plasma membrane increases osmotic membrane water permeability up to about 50-fold compared to the lipid bilayer [4].

From a structural point of view, AQPs usually possess a tetrameric organization in membranes. Each monomer, functioning independently, is quite small, about 30 kDa, and comprises six transmembrane *α*-helical segments, two half helices and five connecting loops of variable length, with the N- and C-terminal domains sited in the cytoplasm. The mechanism allowing AQPs to facilitate water (or glycerol) transport involves an aqueous pore; however, the conveyance of protons is avoided, thus hindering the dissipation of proton gradients [1].

Based on sequence homologies, AQPs could be divided in two groups, the mainly water-permeable AQPs (AQP1, AQP2, AQP4, AQP5, and AQP8) and the mainly glycerol-permeable aquaglyceroporins (AQP3, AQP7, AQP9, and AQP10) [5].

Besides water and glycerol, a variety of metabolically important small uncharged solutes were identified as AQP substrates [6], that is, carbon dioxide [7, 8], nitric oxide [9, 10], lactic acid [11], and metalloids [6, 12]. In the last decade, it has been demonstrated that also hydrogen peroxide (H_2_O_2_) can be carried by some AQP members in several living organisms [13, 14]. Since H_2_O_2_ is considered a second messenger in redox signaling, new interest is rising on the physiological role of H_2_O_2_ channeling through AQP [15]. Recent data from our laboratory demonstrated the capacity of specific AQP isoforms (AQP8 and AQP3) to channel NADPH oxidase- (Nox-) produced hydrogen peroxide across plasma membrane in leukaemia cells, affecting downstream redox signaling pathways linked to leukaemia cell proliferation [16].

## 3. AQP Expression and Physiological Functions 

Cells need to transfer water molecules from inside to outside the plasma membrane and vice versa in order to fulfill a multitude of essential processes, such as the delivery of nutrient substances to and the removal of waste molecules from tissues, the regulation of cell volume, the secretion of fluids from glands, and the migration and proliferation of cells; thus, the distribution of AQPs in different tissues and organs gives an indication on the many functions exerted by these channel proteins. AQPs are particularly abundant in cells appointed to transport fluids, for example, endothelia and epithelia of numerous organs, but unexpectedly also in some cell types not possessing an evident role in fluid transport, such as adipocytes or erythrocytes.

Kidney presents eight of the AQP members; five of them are involved in water absorption and in maintenance of body water homeostasis [17]. AQPs play also an important role in transepithelial fluid secretion in many exocrine glands (airway submucosal and salivary) and further secretory epithelia [18]. Two major isoforms of AQP are highly expressed in the central nervous system, that is, AQP4 and AQP1, whereof AQP4 is particularly in astrocytes, and are involved in the control of volume and water balance of brain and in neuroexcitation and neuroinflammation [19]. In the eye, all the thirteen human AQPs have been found, identified at mRNA level and/or protein level. The main AQP functions in ocular tissues are keeping of corneal and lens transparency, regulation of intraocular pressure, hydration of ocular surface, and transduction of visual signals [20, 21]. AQP3 has been detected in epidermal keratinocytes, where it is involved in skin hydration and is a key player in epidermal proliferation [22].

AQP isoforms are also expressed in blood cells. In macrophages and T lymphocytes, AQP3 facilitates hydrogen peroxide uptake, essential for cell migration toward chemokines [23]. AQP1 and AQP3 expression in erythrocytes have been used for determining blood group: AQP1 is the antigen of the Colton antigen system [24], whereas AQP3 is the antigen of the GIL blood group [25].

Among several unexpected AQPs roles is their involvement in cell migration noticed in many cell types: in this process, AQPs promote transient local water transport [26].

The aquaglyceroporins AQP7 and AQP9 are expressed in the plasma membrane of adipocytes, where these proteins ensure the physiological plasma membrane glycerol permeability [27].

## 4. Polyphenol Modulation of Aquaporin Functions 

### 4.1. Brain

Many brain diseases, such as traumatic brain injury, stroke, brain tumors, and inflammation, result in cerebral edema, an excess of fluid accumulation due to a dysfunction of brain osmotic homeostasis. Water accumulation produces brain swelling [28] and the displacement of water into peripheral blood. These events are accompanied with a progressive rise in intracranial pressure, causing brain ischemia and death [29].

Two classes of cerebral edema have been traditionally considered: cytotoxic, consisting in intracellular water accumulation established in the presence of intact blood-brain barrier, and vasogenic, characterized by blood-brain barrier disruption [30]. However, the recent knowledge about cellular and molecular events indicates this subdivision as a simplified view of a more complicated pathological process, suggesting three major types of the edema build-up phase: anoxic, ionic, and vasogenic edema [31]. Nevertheless, many papers currently refer to the classical subdivision. Studies performed in AQP4-null mice provided the first evidence that AQP4 is deeply involved in brain edema [32]. It has been observed that, in the early phase of cytotoxic edema, AQP4 facilitated edema fluid formation, while the rate of edema fluid elimination was increased by AQP4 in vasogenic brain edema [29]. No efficient treatment is currently available to prevent or limit edema formation or expansion in brain injuries; thus, the discovery of AQP involvement could be useful in the development of new agents able to counteract the edema process. Polyphenols involved in brain pathologies are summarized in Table 1.

Curcumin, a yellow phenolic pigment extracted from the rhizome of* Curcuma longa*, is commonly used as curry spice in Asian cooking and in traditional Indian and Chinese medicine. Much evidence has demonstrated that curcumin is involved in multiple targets and different pathways; moreover, it is endowed with pharmacological safety and enhanced bioavailability, thus being a promising natural compound for the treatment of many human diseases. In particular, recent studies have indicated that curcumin affects a variety of ion channels and transporters [33] and limits neuroinflammation and neurological injury in experimental models of Alzheimer's disease, ischemic stroke, and subarachnoid hemorrhage [34]. Based on these observations, it has been hypothesized that curcumin may limit the development of cerebral edema possibly by reducing AQP4 expression.

In primary astrocyte cultures obtained from the cerebral cortices of mice, Laird and coworkers [36] showed that clinically achievable doses of curcumin attenuated pericontusional AQP4 expression in a way corresponding to the reduction in cerebral edema following traumatic brain injury. Since curcumin reduced also acute IL-1*β* expression in pericontusional astrocytes and attenuated IL-1*β*-induced AQP4 expression in cultured astrocytes, the authors suggested that IL-1*β* may promote cerebral edema* via* the regulation of AQP4. These findings suggest that curcumin may represent a clinically safe AQP4 inhibitor, although the mechanism(s) underlying this potentially beneficial effect remained still unresolved.

In a rat model of hypoxic-ischemic brain damage [35], morphological changes and edema in the brain were observed, with an increased NOS activity and AQP-4 expression in the hippocampus. The authors showed that curcumin treatment partially reversed brain edema and morphological changes as well as hypoxic-ischemic-induced increase in NOS activities and AQP-4 expression. Thus, it is suggested that this phenolic compound may protect astrocytes by downregulating AQP4 and could be considered a promising nutraceutical compound to treat hypoxic-ischemic brain damage.

Also pinocembrin, one of the most abundant flavonoids in propolis, has been reported to protect the rat brain against ischemia injury. In a model of focal cerebral ischemia induced in rats by the middle cerebral artery occlusion, Gao and coworkers [39] demonstrated that pinocembrin alleviated neuronal apoptosis, edema of astrocytic end-feet, and deformation of endothelial cells and capillaries. The results indicate that pinocembrin exerts its protective role by inhibiting both the inflammatory cascade and the AQP4 expression. However, the direct action of pinocembrin on AQP4 expression needs to be confirmed by further investigations.

Spinal cord injury is the consequence of primary and secondary injury events. A physical injury to the spinal cord deriving from the laceration, contusion, and compression of the neural tissues represents the primary event. Secondary injury consists of inflammation processes and further damage following the primary injury [48]. Many pathological processes might be involved in spinal cord injury, such as edema, hypoxia, local ischemia, free radicals production and lipid peroxidation, and apoptosis [49]. A spinal cord injury-induced AQP4 overexpression has also been shown [37]. In a rat acute spinal cord injury model, Zu et al. [37] evaluated the effect of curcumin on the motor function and spinal cord edema, showing that a single dose of curcumin administered* via* the intraperitoneal route at 30 min after acute spinal cord injury was able to moderately improve the motor dysfunction and tissue repair, to attenuate spinal cord edema, and to inhibit the overexpression of AQP4. Authors also observed that during spinal cord injury the JAK/STAT signaling pathway was unusually activated, and further experiments showed that the curcumin protective effect was associated with the inhibition of this pathway. It is concluded that curcumin is a potential compound to be used in the treatment of spinal cord injuries.

Among the dietary polyphenolic compounds, epigallocatechin gallate, the most abundant green tea catechin, is known for its antioxidant action and free radical scavenging effect, but recent evidence has shown many additional actions for catechins and their metabolites [50]. In particular, epigallocatechin gallate has been reported to attenuate neuronal apoptosis and to improve locomotor function after spinal cord injury in rats [51].

Since spinal cord injury is associated with edema and changes in AQP4 expression, Ge and coworkers [38] investigated the potential antiedema effect of epigallocatechin gallate and its underlying mechanism on a rat model of acute spinal cord injury. The results show that epigallocatechin gallate could significantly reduce spinal cord water content, and this effect was ascribed to the marked downregulation of AQP4 expression induced by the epigallocatechin gallate treatment.

As previously reported, AQP4 is the principal water channel expressed in astrocytes throughout the central nervous system, but other members of the AQP family are present in the brain. In the apical surface of the choroid plexus, the main AQP isoform expressed is AQP1 [52]. The choroid plexus is a secretory epithelium located within the brain, involved in the production and secretion of cerebrospinal fluid. In this context, AQP1 plays an important role in the facilitation of water transport across the cells, thus contributing to cerebrospinal fluid formation and secretion. Recent studies have shown that, after severe brain injury, AQP1-null mice showed reduced intracranial pressure and improved survival, compared to wild-type mice [53]. Thus, it is conceivable that selective blockage of AQP1 activity or expression in the choroid plexus would counteract the elevated intracranial pressure accompanying many brain disorders, such as traumatic brain injury, hydrocephalus, stroke, systemic hyponatremia, acute cerebral edema, and hypertension [54]. According to this hypothesis, the identification of selective AQP1 inhibitors could be useful for therapeutic treatment of these disorders. Nabiuni et al. [40] have demonstrated that the phenolic pigment curcumin can decrease the AQP1 level in choroidal epithelial cells from Wistar rats in a dose-dependent manner. It was suggested that this dietary phenolic compound might regulate water homeostasis in the central nervous system, thus reducing intracranial pressure under pathophysiological conditions.

Systemic lupus erythematosus represents a complex, autoimmune, and heterogeneous pathologic condition that includes edema, inflammation, and brain atrophy [55]. Besides chronic inflammation, neuropsychiatric disease and cognitive dysfunctions occur in the majority of patients, making this pathology a devastating disorder.

Since the knowledge about this human disease is very limited, a number of animal models have been developed. One of the widely used and accepted lupus erythematosus animal models is MRL/*lpr* mice [55]. Given the broadly documented pharmacological activities of curcumin, including neuroprotective effects, a study was conducted to test whether this compound might improve the lupus-induced central nervous system injury [41]. Results showed that curcumin treatment of MRL/*lpr* lupus mice compared to controls worsened brain atrophy and increased the edematous cells size, and, in line with these observations, an increase in mRNA level and protein expression of AQP4 occurred in comparison to controls. Furthermore, also the neurological performance of curcumin-treated MRL/*lpr* mice was worsened compared to the untreated counterparts. Diverging from other results reported in the literature, worsening of disease was observed in MRL/*lpr* mice in the presence of curcumin, and thus caution should be adopted in the use of curcumin as therapeutic agent for the treatment of lupus erythematosus, provided that the validity of these findings is confirmed in human patients.

### 4.2. Eye

AQPs are pores that facilitate water movement through cellular membranes, playing a crucial role in the eye physiology by maintaining water homeostasis [21]. The retina constitutes the key light-sensitive tissue of the eye and it is made up of several layers of neurons and glial cells. The importance of AQP water channels in retina cells is highlighted by the observation that mRNAs of all the 13 known AQP isoforms (from AQP0 to AQP12) are expressed in the human retina [41]. AQP4 is the most abundant isoform expressed in the retina [56], especially in astrocytes and in Müller glial cells, where it is localized in the end-feet membranes [21].

Diabetic retinopathy is a serious complication of diabetes mellitus that can eventually lead to blindness. It is the result of microvascular retinal changes: during the first stage blood vessels swell and leaked fluid produces edema, as the disease progresses new vessels proliferate, impairing vision and ultimately detaching retina. The reasons of these changes are due to prolonged hyperglycemia, which leads to increased ROS generation in retina, establishing an oxidative stress condition associated with cellular inflammation and release of inflammatory cytokines, and to the hyperglycemia-induced formation of advanced glycation end products.

Since it has been shown that AQP4 is involved in the generation of diabetic retinal edema through a redistribution of its expression [57], AQP4 has been suggested as a novel therapeutic target for the treatment of diabetic retinopathy.

Quercetin is flavonol that can be found in many vegetables, and it is one of the major bioflavonoids in the human diet. Besides its well-known activity as antioxidant and phase 2 enzyme inducer [58], quercetin has been found also to be efficacious in many ocular diseases [42].

In streptozotocin-induced diabetic rats, Kumar and coworkers [42] observed that quercetin treatment exerted remarkable neuroprotective effects on diabetic retina. They reported that quercetin-treated rats exhibited a positive modulation of antioxidant enzymes and improved GSH level. Moreover, quercetin treatment caused the inhibition of cytokine release and the trigger of apoptosis through the prevention of microglia activation. Finally, quercetin treatment inhibited AQP4 expression in Müller cell end-feet and perivascular space, thus preventing retinal edema.

Hesperetin is a flavanone glycoside contained in many fruits of* Citrus* genus, and it has been reported to be effective as radical scavenger and to improve the protective cellular antioxidant enzyme activities from exogenous oxidative stress [43, 59]. Furthermore, hesperetin has been found to exert neuroprotective effects [43]; thus, it could be considered for the treatment of diabetic retinopathy, since oxidative stress, inflammation, and neurovascular complications are implicated in this pathology. The effect of hesperetin was studied in the streptozotocin-induced diabetic rat's model, evaluating histological retinal changes after a 24-week hesperetin treatment [44]. In hesperetin-treated rats, GSH content was restored to control level, and antioxidant enzyme activities were positively regulated. Furthermore, hesperetin-treated retina showed an inhibition of both the activity of caspase-3 and the production of glial fibrillary acidic protein, along with lower inflammatory cytokines levels compared to control rats [44]. In particular, hesperetin-treated rats showed AQP4 downregulation at Müller cell end-feet.

Therefore, the authors suggested that APQ4 inhibition could be a new therapeutic strategy for diabetic retinopathy and that polyphenols such as quercetin and/or hesperetin could be interesting candidates in such a new approach [42, 44] (see Table 1).

### 4.3. Skin

AQP3 is the most abundant aquaporin present in the skin, and it is expressed in the plasma membrane of epidermal keratinocytes [60]. AQP3 belongs to the subfamily of aquaglyceroporins, since it is able to transport both water and glycerol. Being an effective water-retaining osmolyte, glycerol is the major skin humectant in the epidermis and stratum corneum. AQP3-mediated diffusion of glycerol and, consequently, of water, is a fundamental mechanism for skin hydration. AQP3-deficient mice model exhibits reduced elasticity and hydration in skin, along with mechanical and biosynthetic defects [61]. In fact, it has been recently reported that a reduction in skin glycerol content results in impaired epidermal proliferation [62], since glycerol is an important metabolite of skin cells, being involved in ATP production and in membrane lipids biosynthesis. Further, overexpression of AQP3 induced keratinocyte proliferation and epidermal hyperplasia [63]. In contrast, AQP3-knockout mice have been shown to be resistant to tumorigenesis as a result of reduced glycerol content and diminished ATP biosynthesis [64]. Indeed, AQP3 plays a pivotal role in the regulation of glycerol-related metabolism in the epidermis; therefore, AQP3 inhibition has been proposed as new therapeutic strategy in many hyperproliferative skin disorders (see Table 1) such as psoriasis, atopic dermatitis, and skin carcinogenesis [45, 63, 64].

Resveratrol is a stilbene polyphenol that is found in plants relevant to human diet such as grapes, blueberry, and raspberry. Resveratrol is one the most studied polyphenols, since it is involved in antiproliferative, antioxidant, anti-inflammatory, antiangiogenic, and antimetastatic effects in many different cell lines [65].

By using AQP3 siRNA, Wu and coworkers [45] confirmed that the expression of AQP3 was essentially involved in the proliferation of neonatal normal human epidermal keratinocytes and demonstrated that resveratrol decreased the expression of AQP3 in a concentration-dependent manner, hence inhibiting cell proliferation. Authors have also demonstrated that the inhibitory effect of resveratrol on the AQP3 expression was dependent on the inhibition of the ERK phosphorylation through a mechanism involving the upregulation of SIRT1 and the aryl hydrocarbon receptor nuclear translocator molecules.

Ultraviolet light, both UVA and UVB, can penetrate the atmosphere and reach the skin. UV irradiation is able to produce a series of harmful effects on the human skin, spanning from sunburn to skin cancer [66, 67]. Acute UV irradiation can also cause human skin dehydration that might be attributed, at least in part, to AQP3 downregulation, as demonstrated in human keratinocytes [68]. Thus, a reduction in the negative effect of UV light on the expression of AQP-3 could have beneficial effect on skin photoprotection.

Chrysin is a natural flavonoid, extracted from honey and propolis and also present in various edible plants [69]. Recent studies have revealed antioxidant, anti-inflammatory, anticancer, and antimicrobial activities of chrysin [70]. The putative protective effect of chrysin on the UV-produced damage in the skin was studied by Wu and coworkers in human keratinocytes cell lines (HaCaT cells) [46]. Authors confirmed that UVB is able to downregulate AQP-3 expression in HaCaT keratinocytes and observed that the pretreatment with chrysin exerted a protective effect on AQP3 downregulation in a dose-dependent manner. Furthermore, chrysin protected keratinocytes against apoptosis, ROS overproduction, and COX-2 induction caused by physiological doses of UVA and UVB radiation. Therefore, the potential use of chrysin, a natural compound in common foods, could be useful for the prevention of UV-induced deleterious effects on human skin.

## 5. Adipocyte Metabolism

The aquaglyceroporin AQP7 is expressed in the plasma membrane of adipocytes [4, 27]. Adult AQP7-deficient mice show both elevated intracellular glycerol content and increased activity of adipose glycerol kinase as compared to mice at young age. This glycerol kinase upregulation enhances the esterification of glycerol and accelerates triglyceride accumulation in adipocytes, thus causing adipocyte hypertrophy [27]. These findings suggest that adipocyte glycerol permeability could regulate adipocyte metabolism, supporting the hypothesis that modulation of adipocyte AQP7 function could be a preventive/therapeutic strategy for obesity and metabolic syndrome (Table 1).

It has been reported that apple polyphenols exhibit antihyperglycemic, antihyperlipidemic, and anti-inflammatory properties [71–73]. Indeed, apples are a rich source of polyphenols, which consist of a complex mixture of chlorogenic acid, phloridzin, quercetin, catechin, epicatechin, procyanidin, and rutin, among other compounds [74]. Furthermore, apple polyphenols have been reported to have an inhibitory effect on adipose tissue formation in Wistar rats [75] and to reduce triglyceride absorption by the inhibition of pancreatic lipase activity in mice and humans [76]. A study conducted on diet-induced obesity in Wistar rats evidenced that the supplementation with apple polyphenols prevented adiposity increase by inhibition of adipocyte hypertrophy [47]. Among the other effects, apple polyphenols intake led to an increase of AQP7 expression in epididymal adipocytes, and this raise could explain the increased glycerol release noticed in the* ex vivo* lipolysis experiments and the smaller size of the adipocytes observed in rats supplemented with apple polyphenols [47].

## 6. Conclusive Remarks

This review presents an overview of experimental data reporting the strong implication of AQPs in several pathologies and diseases, and, for the first time to our knowledge, highlights the involvement of bioactive compounds, like polyphenols, in the prevention and/or reduction of such pathologies. In most cases, polyphenols act by the reduction of AQP levels or AQP overexpression due to disease; less frequently, these molecules reverse the downregulation of AQPs bringing levels to normal values.

The mechanism of action is still not clear, and certainly the beneficial effect cannot be ascribed only to antioxidant properties of polyphenols; therefore, further studies are required.

Nevertheless, AQP modulation represents an innovative potential therapeutic strategy. A challenge in AQP-based therapeutics is the identification of compounds acting as AQP inhibitors or being able to increase AQP expression. Polyphenols, largely occurring in plant-derived foods, could represent a healthy and intriguing opportunity in the prevention and treatment of diseases where AQPs are involved. Clinical trials on humans would be useful to corroborate the hypothesis of a possible AQP-based polyphenol treatment.

## Figures and Tables

**Table 1:** Involvement of AQP isoforms and polyphenols in some pathologies.

Pathology	AQP isoform involved	Active polyphenol	Action	Observed effects	References
Brain edema	AQP4	Curcumin	Downregulation of AQP4 expression	Reduced neuroinflammation and neurological injury	[33–35]

Traumatic brain injury	AQP4	Curcumin	AQP4 inhibition	Reduced cerebral edema	[36]

Spinal cord injury	AQP4	Curcumin	Inhibition of AQP4 overexpression	Improvement of motor dysfunction and attenuation of spinal cord edema	[37]

Spinal cord injury	AQP4	Epigallocatechin gallate	Downregulation of AQP4 expression	Improvement of locomotor function and reduction of spinal cord edema	[38]

Focal cerebral ischemia	AQP4	Pinocembrin	Reduced AQP4 expression	Reduced neuronal apoptosis and astrocytic end-feet edema	[39]

Brain injury	AQP1	Curcumin	Reduced AQP1 expression	Reduction of intracranial pressure	[40]

Lupus erythematosus	AQP4	Curcumin	Increased AQP4 expression	Worsening of brain atrophy and increase of the edematous cell size	[41]

Diabetic retinopathy	AQP4	Quercetin and hesperetin	Downregulation of AQP4 expression	Neuroprotection, prevention of retinal edema, restoring of GSH normal levels, and improvement of antioxidant enzyme activities	[42–44]

Skin disorders	AQP3	Resveratrol	Reduced AQP3 expression	Inhibition of keratinocyte proliferation	[45]

UV-produced skin damage	AQP3	Chrysin	Inhibition of AQP3 downregulation	Protection of keratinocytes against apoptosis and ROS overproduction	[46]

Obesity	AQP7	Apple polyphenols	Increased AQP7 expression	Inhibition of adipocyte hypertrophy	[47]
